# Effects of Pressure on Exciton Absorption and Emission in Strongly Quantum-Confined CsPbBr_3_ Quantum Dots and Nanoplatelets

**DOI:** 10.1021/acs.jpcc.3c08029

**Published:** 2024-01-31

**Authors:** Chih-Wei Wang, Ebube E. Oyeka, Alison B. Altman, Dong Hee Son

**Affiliations:** † Department of Chemistry, Texas A&M University, College Station, Texas 77843, United States; ‡ Department of Physics and Astronomy, Texas A&M University, College Station, Texas 77843, United States; § Center for Nanomedicine, Institute for Basic Science and Graduate Program of Nano Biomedical Engineering, Advanced Science Institute, Yonsei University, Seoul 03722, Republic of Korea

## Abstract

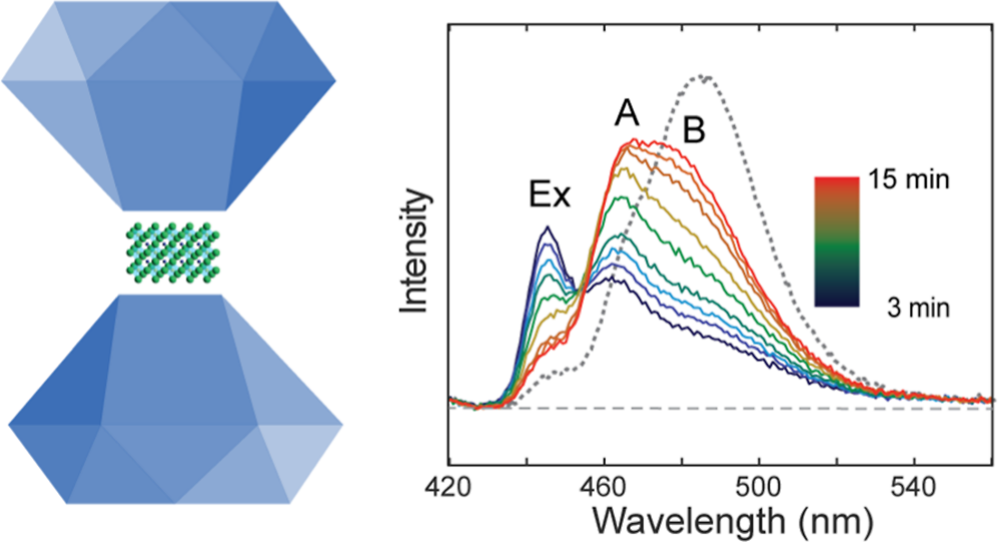

Soft lattices of metal halide perovskite (MHP) nanocrystals (NCs) are considered responsible for many of their optical properties associated with excitons, which are often distinct from other semiconductor NCs. Earlier studies of MHP NCs upon compression revealed how structural changes and the resulting changes in the optical properties such as the bandgap can be induced at relatively low pressures. However, the pressure response of the exciton transition itself in MHP NCs remains relatively poorly understood due to limitations inherent to studying weakly or nonconfined NCs in which exciton absorption peaks are not well-separated from the continuum interband transition. Here, we investigated the pressure response of the absorbing and emitting transitions of excitons using strongly quantum-confined CsPbBr_3_ quantum dots (QDs) and nanoplatelets (NPLs), which both exhibit well-defined exciton absorption peaks. Notably, the reversible vanishing and recovery of the exciton absorption accompanied by reversible quenching and recovery of the emission were observed in both QDs and NPLs, resulting from the reversible pressure modulation of the exciton oscillator strength. Furthermore, CsPbBr_3_ NPLs exhibited irreversible pressure-induced creation of trap states at low pressures (∼0.1 GPa) responsible for trapped exciton emission that developed on the time scale of ∼10 min, while the reversible pressure response of the absorbing exciton transition was maintained. These findings shed light on the diverse effects the application of force has on the absorbing and emitting exciton transitions in MHP NCs, which are important for their application as excitonic light emitters in high-pressure environments.

## Introduction

The effects of applying external pressure on the structure of semiconductor nanocrystals (NCs) and the accompanying changes in their properties including optical, electronic, and phonon properties have been extensively investigated for decades.^1−3^ In NCs, the pressure-dependent structure and the rates of the structural change differ from the bulk phase due to the surface contribution influenced by the ligand and NC shape in determining the phase equilibrium and kinetics,^4−6^ providing additional means to pressure-modulate the semiconductor properties.

Recently, research on metal halide perovskite (MHP) NCs under high pressure has revealed several distinct behaviors that reflect the soft lattice and facile structural transformation of the MHP NCs. For instance, a pressure-induced structural change altering the interband transition properties was observed in MHP NCs at significantly lower pressures than in various II–VI semiconductor NCs that have been extensively studied. Specifically, in II–VI semiconductor NCs, the wurtzite-to-rock salt phase transition resulting in the direct-to-indirect bandgap transition is reported to occur at 3.6–5 GPa for CdSe NCs, 6–8 GPa for CdS NCs,^7^ and >10 GPa for ZnS NCs.^8,9^ In comparison, in CsPbBr_3_ NCs, inversion of the direction of the bandgap shift is reported to occur in the range of 0.7–1.5 GPa attributed to an isostructural phase transition associated with the change in the mode of deformation in [PbBr_6_]^4–^octahedra.^10−12^ When organic cations replace Cs^+^ cation sites, e.g., in FAPbBr_3_ (FA = CH_3_NH_3_^+^), even amorphization of the lattice was observed at relatively low pressures (2–4 GPa) that may reflect the more flexible nature of the organic ions.

The observation of the trapped exciton PL in MHP NCs under relatively low pressure also indicates that the lattice of MHP can deform readily, although the appearance of the trapped exciton PL varies significantly among NCs of different compositions and structures. In CsPbCl_3_ NCs of nanosheet form, PL from a self-trapped exciton (STE) was observed above 2.7 GPa following the quenching of the exciton PL at 1.65 GPa.^13^ In Cs_4_PbBr_6_ NCs, often referred to as bulk 0D NCs due to the isolated [PbBr_6_]^4–^ octahedra in the lattice, the PL from the STE was observed above ∼3 GPa.^14^ The PL from the STE in Cs_4_PbBr_6_ NCs was also accompanied by the emergence of a new absorption peak attributed to the absorbing STE transition that was not observed in CsPbCl_3_ NCs, suggesting a relatively high density of STE states in this system. In the 2-dimensional (2D) layered organic–inorganic hybrid perovskites (A_2_PbX_4_, A = organic cation, X = halide), the effect of pressure on the PL from exciton and STE is more complex, reflecting the higher structural and chemical diversity of this family of MHPs.^15−17^

So far, the pressure effects on the optical absorption and PL of colloidal MHP NCs have predominantly been performed in relatively large NCs whose dimensions are larger than the exciton Bohr diameter. Therefore, except for the layered 2D hybrid systems, the exciton that dictates the optical properties of these NCs could not be well resolved from the overlapping continuum interband transition in the absorption spectra of MHP NCs. For this reason, while the earlier studies in weakly and nonconfined 3-dimensional MHP NCs revealed the pressure-dependent (bulk) bandgap, it was difficult to unambiguously determine the pressure effect on the exciton transition itself.

In this work, we investigated the effects of pressure on both absorbing and emitting exciton transitions in strongly quantum-confined CsPbBr_3_ NCs, where a distinct exciton absorption peak is well separated from the continuum transition.^18,19^ We used 3.7 nm cube-shaped CsPbBr_3_ quantum dots (QDs) and 2 nm thick CsPbBr_3_ nanoplatelets (NPLs), whose confined dimension is smaller than the exciton Bohr diameter of CsPbBr_3_ (∼7 nm). QDs and NPLs are among the most common morphologies of colloidal MHP NCs, each representing 0D and 2D semiconductor NCs. Interestingly, we observed a vanishing exciton absorption peak in both CsPbBr_3_ QDs and NPLs as the pressure increased, which was not resolvable in the earlier studies in large NCs. Our results indicate that varying the pressure not only shifts the exciton transition energy but also reversibly turns off and on the exciton absorption. In addition, CsPbBr_3_ NPLs exhibited only the “trapped” exciton PL with a large Stokes shift (>250 meV) even at the lowest pressure (0.1 GPa) in an irreversible manner, in contrast to the exciton absorption exhibiting a reversible pressure response of the “free” exciton. The time-dependent PL measurement on CsPbBr_3_ NPLs at 0.1 GPa suggested the irreversible pressure-induced creation of trap states occurring on a time scale of several minutes. Such behavior contrasts with that of CsPbBr_3_ QDs that exhibit absorbing and emitting transitions from the same exciton under pressure and may reflect the higher susceptibility of thin NPLs to the pressure-induced structural deformation that creates the trap states. The results from this study reveal the different pressure effects on the absorbing and emitting exciton transitions in MHP NCs and how they vary with their structural details that will be valuable for utilizing these materials as light emitters in environments that exert pressure or stress.

## Methods

### Synthesis of Materials and Characterization

CsPbBr_3_ QDs were synthesized following a method utilizing the thermodynamic equilibrium-based size control that enables precise control of the size in a strongly quantum-confined regime.^18^ Cs-oleate was prepared by mixing Cs_2_CO_3_, oleic acid (OA), and 1-octadecene (ODE) in a trineck round-bottomed flask. The solution was degassed and kept under N_2_ at 120 °C. In a separate round-bottomed flask, PbBr_2_, ZnBr_2_, ODE, OA, and oleylamine (OAm) were combined and degassed at 120 °C until all solids were dissolved. Subsequently, the flask was purged with N_2_ before being raised to the temperature needed for the reaction. Cs-oleate was injected swiftly into the reaction flask before quenching with an ice bath. The crude solution was allowed to stand on the benchtop under ambient conditions until no precipitate was observed in the solution. The NCs were washed with acetone and hexane before being dispersed in hexane for further use.

2 nm thick CsPbBr_3_ NPLs were synthesized using the previously reported method by Dong et al.^20^ In brief, a round-bottom flask containing PbBr_2_, CuBr_2_, CoBr_2_, OA, OAm, and ODE was degassed at 120 °C. After purging with N_2_, the temperature was raised to 200 °C for 5 min. The mixture was then cooled to room temperature with a water bath, and Cs-oleate was added. The formation of NPLs was initiated with the addition of acetone. The reaction was allowed to proceed for 1 min, and the produced NPLs were collected via centrifugation. The NPLs were then washed with methyl acetate and hexane before being suspended in hexane. Afterward, 100 μL of 0.02 M didodecyldimethylammonium bromide in toluene was added to the NPL solution and stirred for 30 min to enhance the PL. Excess ligands were removed by repeating the washing procedure. A transmission electron microscope (FEI TECNAI G2 F20 ST) was used to confirm the size and morphology of the NCs by transmission electron microscopy (TEM). The NC samples were prepared by drop-casting the NC solution onto a TEM grid. The grid was then dried in vacuo before the measurement.

### High-Pressure Sample Preparation in a Diamond Anvil Cell and Spectroscopic Measurements

A BX-80 diamond anvil cell (DAC) with 80° openings was equipped with two 500 μm culet Type IIa (100)-oriented standard cut diamonds seated on 60° conical tungsten carbide supports. A laser micromachining system from DACTools was used to drill a 300 μm hole into a Re gasket that had been preindented to a final thickness of ∼70 μm. The gasket was centered on the diamond, and a ruby sphere (3600 ppm of Cr^3+^: Al_2_O_3_, BETSA) was placed into the cylindrical sample space defined by the gasket. A solution of the sample dispersed in either an ODE or silicone oil (from Sigma-Aldrich) was dropped via a microsyringe into the sample space, and the cell was closed to create a seal, with the silicone oil or the ODE solvent acting as the pressure-transmitting medium. Pressure in the DAC was measured using the R1 emission line of the ruby.^21,22^ Both the absorption and PL spectra of the sample in the DAC were measured using a home-built optical microscope composed of a 10× objective (Nikon) and a tube lens (Nikon) as well as a pair of charge-coupled device (CCD) cameras and CCD spectrometers for imaging and recording the spectra. A 405 nm diode laser was used for excitation in all of the PL experiments. A deuterium-halogen lamp (UV/vis-ISS, Ocean Optics) was used for all of the absorption measurements. A CCD spectrometer (QE 65 Pro, Ocean Optics) was used to record all of the spectra. Time-resolved PL spectra of the NCs were measured by using a pulsed 405 nm diode laser (LDH P-C-405, PicoQuant) and a time-correlated single photon counting system (PicoHarp 300, PicoQuant).

## Results and Discussion

**Figure 1:**
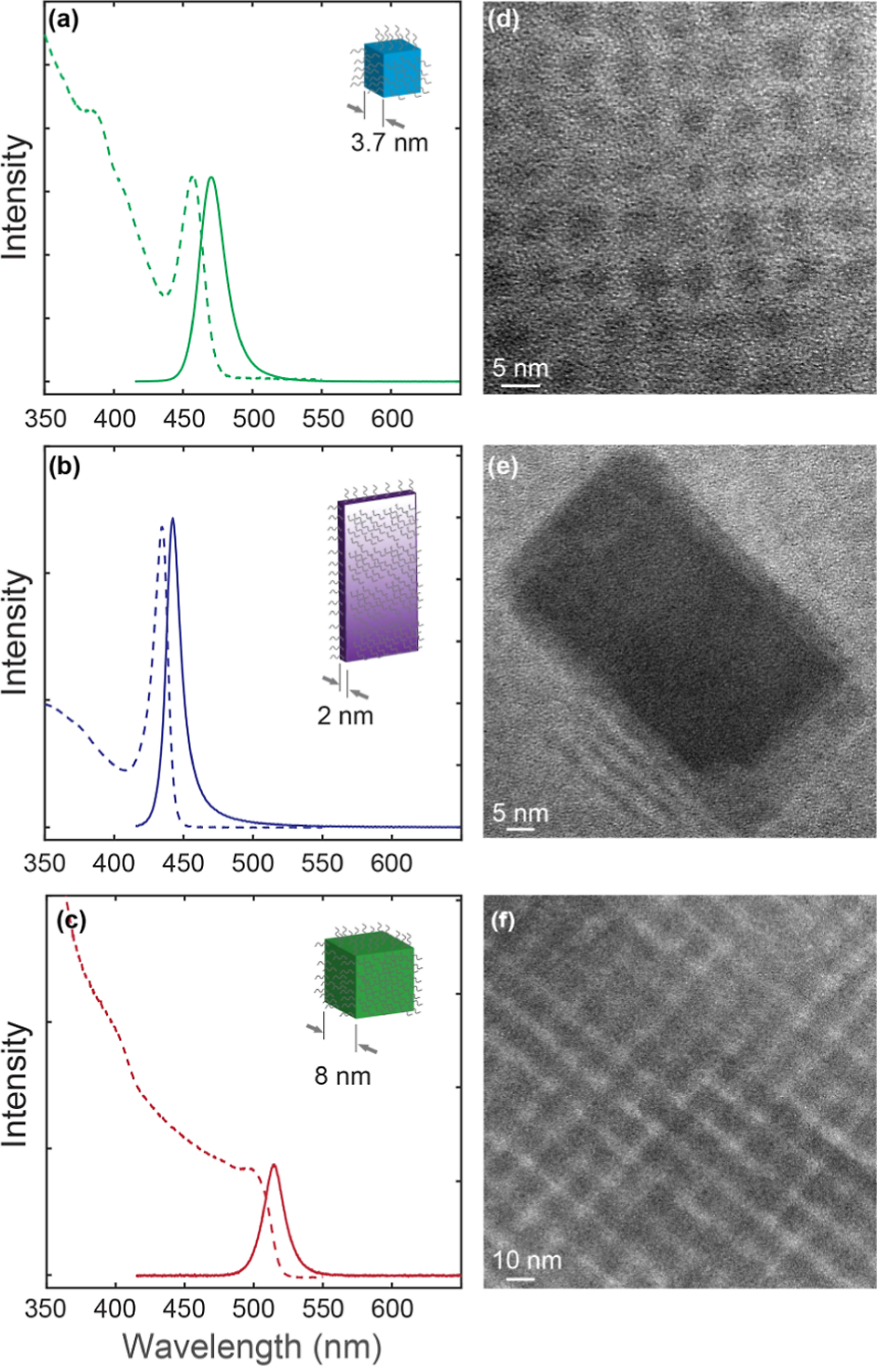
(a–c) Solution-phase absorption (dashed) and PL (solid) spectra of CsPbBr_3_ NCs. (a) 3.7 nm CsPbBr_3_ QDs, (b) 2 nm thick CsPbBr_3_ NPLs, and (c) 8 nm weakly confined CsPbBr_3_ QDs that do not show a well-isolated exciton absorption peak. (d–f) TEM images of 3.7 nm QDs, 2 nm thick NPLs, and 8 nm QDs.

To investigate the effects of the pressure on the exciton properties in strongly quantum-confined CsPbBr_3_ NCs, 3.7 nm QDs and 2 nm thick NPL that have a confined dimension smaller than the exciton Bohr diameter of CsPbBr_3_ (∼7 nm) were synthesized, as described in the Methods section. Figure 1a,b shows the solution-phase absorption and PL spectra of CsPbBr_3_ QDs and NPLs under ambient pressure. The TEM images of these NCs are shown in Figure 1d,e. The edge length and thickness of the QD and NPL correspond to 6 and 3 units of [PbBr_6_]^4–^ octahedra, respectively. The surfaces of the QDs and NPLs are passivated with organic ligands to provide colloidal stability in the solvent and pressure-transmitting medium. Because of the strong quantum confinement, CsPbBr_3_ QDs and NPLs exhibit well-defined and narrow exciton absorption peaks, enabling the monitoring of the spectral evolution of both the absorbing and the emitting exciton transitions clearly under pressure. For comparison, the absorption and PL spectra of weakly confined NCs (8 nm) that do not show a well-defined exciton absorption peak are shown in Figure 1c.

To assess the pressure-dependent optical properties of the NCs, we loaded solutions of each NC into a DAC. For each experiment, the cell was barely closed to ensure the lowest possible pressure at the onset in a sealed sample environment. A gradual increment in pressure was achieved by gently compressing the cell using 4 hand-turned screws until the absorption and PL peaks vanished. Subsequently, decompression was performed until the maximum possible pressure release was achieved (indicated by the free movement of the screws). Each measurement was conducted in triplicate using the same cell to maintain consistency. To note, although silicone oil or paraffin oil has been more frequently used in the earlier studies of perovskite NCs, we chose ODE as the pressure-transmitting medium as it forms the better dispersion of the NCs compared to that of the silicone oil. We confirmed that the PL spectra observed in ODE are consistent with those in silicone oil and paraffine oil both in terms of the peak position and line width, reflecting the observation that PL spectra are less affected by aggregation (see Figure S2 in the Supporting Information for further details). However, the absorption spectra of samples in silicone oil suffered from distortion of the background attributed to sample aggregation that inhibited reliable measurement of the pressure-dependent absorption spectra, as shown in Figure S3 in the Supporting Information. Thus, to enable complete comparisons of both the PL and absorption spectra, we utilized ODE as the pressure-transmitting medium for all pressure-dependent data presented. Notably, no significant broadening of the R1 emission line for the ruby was observed over the entire pressure regime examined, suggesting that quasi-hydrostatic conditions were maintained throughout the experiments (see Figure S4).

**Figure 2:**
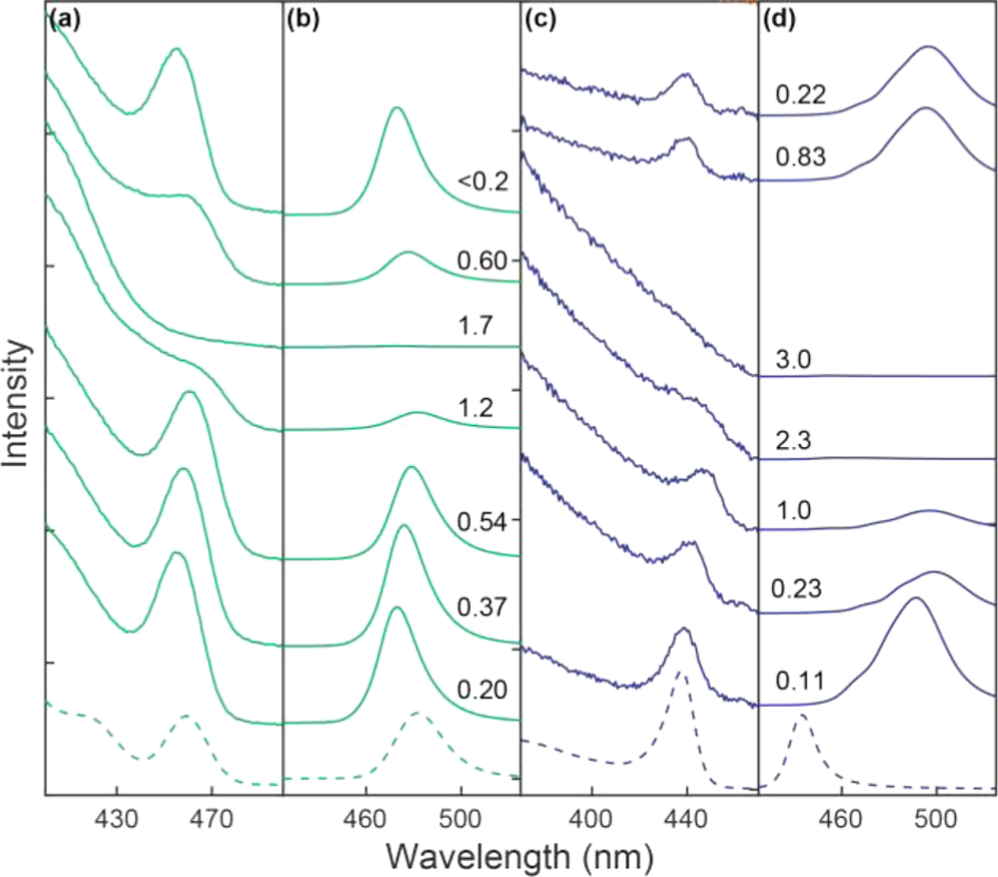
(a,b) Pressure-dependent (a) absorption and (b) PL spectra of 3.7 nm CsPbBr_3_ QDs. (c,d) Pressure-dependent (c) absorption and (d) PL spectra of 2 nm thick CsPbBr_3_ NPLs. The pressure value indicated for each spectrum is in GPa, and an ODE was used as the pressure-transmitting medium. The dashed curves are the ambient pressure spectra.

**Figure 3:**
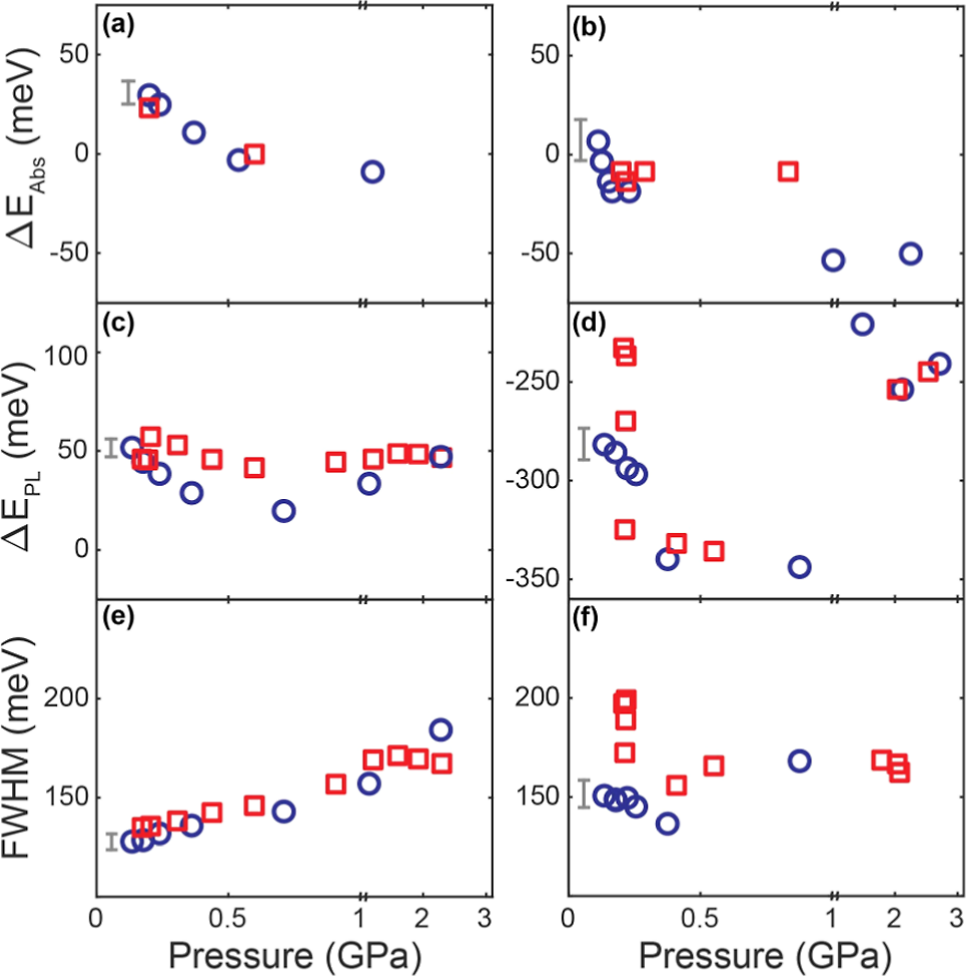
Pressure-dependent peak shift of the exciton absorption (Δ*E*_Abs_), PL (Δ*E*_PL_), and FWHM of the PL during the compression (○) and decompression (□) cycles. (a,c,e) 3.7 nm CsPbBr_3_ QDs. (b,d,f) 2 nm thick CsPbBr_3_ NPLs. Δ*E*_Abs_ and Δ*E*_PL_ are referenced to the peak positions at the ambient pressure. The error bar in each panel represents a typical uncertainty in the measurement below ∼1 GPa and becomes higher above 1 GPa. ODE was used as the pressure-transmitting medium.

In Figure 2, a representative pressure-dependent absorption and PL spectra of CsPbBr_3_ QDs and NPLs measured in a DAC are compared for a few selected pressures in the 0–3 GPa range to provide an overview of the spectral evolution during the compression and decompression cycles (see Figure S1 in Supporting Information for the complete data set). As a reference for the comparison, the spectra of the NCs under ambient pressure are also shown at the bottom of each panel. Since both the exciton absorption and emission peaks disappear between 1 and 2 GPa without new peaks at the higher pressures, the 0–3 GPa range was sufficient to examine the pressure effect on exciton transitions for both QDs and NPLs in this study. The pressure-dependent relative shift of the absorption (Δ*E*_Abs_) and PL (Δ*E*_PL_) peaks referenced to the ambient pressure spectra as well as the pressure-dependent full-width half-maximum (FWHM) PL line width are shown in Figure 3. The error bar shown in each panel of Figure 3 represents a typical uncertainty of the measurement below ∼1 GPa, where the exciton absorption and PL peaks are well-defined. For all the high-pressure spectra reported in Figure 2, the measurements were made after waiting a sufficiently long time (∼20 min) to reach a steady equilibrated spectrum at each pressure. Time-dependent variation of the spectra for a given pressure, which is especially pronounced for the PL spectra of CsPbBr_3_ NPLs at the low-pressure regime (0.1 GPa), will be discussed separately in a later part of the discussion.

In Figure 2a,b, both the absorption and PL peaks of CsPbBr_3_ QDs in a DAC show a small but continuous shift to a longer wavelength as the pressure increases up to ∼1 GPa. Pressure-dependent Δ*E*_Abs_ and Δ*E*_PL_ are plotted in Figure 3a,c. At pressures >∼1 GPa, the distinct exciton absorption peak and PL disappear. Upon decompression, the exciton absorption peak and PL are recovered, indicating the reversible pressure response of both absorbing and emitting exciton transitions. When compared to the spectra at the ambient condition, the absorption and PL spectra show a small blue shift at the first pressure point observed in the DAC, although a further increase of the pressure results in a continuing red shift up to ∼1 GPa with respect to the initial pressure point. Such an initial blue shift has also been reported in an earlier study on larger CsPbBr_3_ QDs.^11^ While its origin remains unclear, we conjecture that a partial rearrangement of the surface ions in the cube-shaped QDs resulting in a slight change in the shape and the exciton confinement potential may contribute to the initial blue shift. Here, we will focus on discussing the pressure-dependent evolution of the absorption and PL spectra upon a change in pressure in the DAC.

A gradual and continuous change of both the absorption and PL peaks and the FWHM PL line width with increasing pressures indicates that the nature of the absorbing and emitting exciton transitions does not change abruptly within the pressure range in which they appear clearly. It is also notable that the vanishing and recovery of the exciton absorption peak are accompanied by the quenching and recovery of the PL. This concomitance suggests that the vanishing of the exciton transition at higher pressures is responsible for the quenching of the PL rather than the enhancement of the nonradiative pathways competing with the radiative recombination of the same exciton under lower-pressure conditions. Although the present study cannot address the structural change in CsPbBr_3_ QDs under pressure, the data clearly show that the higher-pressure, nonemitting QDs do not have a dipole-allowed exciton transition. In an earlier study of larger (12 nm) nonconfined CsPbBr_3_ NCs, a gradual initial decrease of the bandgap followed by an increase that begins at ∼1.2 GPa was reported based on the observation of the shift of the absorption edge and/or PL peak.^12^ The red shift was explained as the result of the isotropic compression of [PbBr_6_]^4–^ octahedra that decreases the bulk bandgap.^9^ The subsequent blue shift was attributed to the changes in the mode of deformation from isotropic compression to tilting of [PbBr_6_]^4–^ octahedra upon further compression that increases the bulk bandgap.^23^ However, it was difficult to determine whether the exciton transition remains or vanishes upon compression in the earlier studies since the exciton absorption peak is not clearly separable from the continuum interband transition. The data in Figure 2a,b clearly show that the effect of pressure on the electronic structure of CsPbBr_3_ QDs is not only altering the bulk bandgap, as the earlier studies have established, but also switching off the well-defined exciton transition, a key characteristic optical feature of the semiconductor QDs.^24^

In certain lead bromide perovskites, the loss of PL under compression was attributed to the pressure-induced loss of long-range order from amorphization of the lattice, e.g., in MAPbBr_3_,^25^ where the defects from the amorphization can potentially quench the PL. However, it is unlikely that defects from the loss of long-range order are responsible for the reversible vanishing and recovery of both the exciton absorption and PL peaks in CsPbBr_3_ QDs occurring at >∼1 GPa. Another possible cause for the PL quenching accompanying the vanishing of the exciton absorption is a direct-to-indirect bandgap transition. Such a transition has been previously observed in CdSe QDs under high pressure, where the exciton absorption peak present in the direct gap structure (wurtzite) became invisible with a phase transition into the indirect gap structure (rock salt).^26^ Although further studies are needed to determine the exact mechanism, the observed pressure-induced switching of the exciton transition has an important implication when using MHP NCs as excitonic photon emitters under high-pressure conditions.

Compared to those of CsPbBr_3_ QDs, the pressure-dependent absorption and PL spectra of CsPbBr_3_ NPLs in Figure 2c,d show quite different responses to the change in pressure. The exciton absorption peak of CsPbBr_3_ NPLs exhibits a gradual red shift with increasing pressure until it disappears at >∼2 GPa in a qualitatively similar manner to that of CsPbBr_3_ QDs. In contrast, the PL of CsPbBr_3_ NPLs is very broad and largely red-shifted (230–350 meV) with respect to the PL under ambient pressure in the entire pressure range of this study. Clearly, the PL under pressure originates from a state that is different from the absorbing exciton transition. The PL spectra under pressure have multiple peaks that red-shift with increasing pressure gradually. The quenching of the PL coincides with the disappearance of the exciton absorption peak, which recovers on the decompression cycle, analogous to CsPbBr_3_ QDs. However, the PL spectrum at the end of the decompression cycle still shows a largely red-shifted peak from that observed at ambient pressure, as will be discussed further in detail later. The pressure dependence of Δ*E*_Abs_, Δ*E*_PL_, and FWHM of the PL from CsPbBr_3_ NPLs is shown in Figure 3b,d,f. Below ∼0.3 GPa, where the pressure-dependent peak shift is the largest, Δ*E*_PL_ shifts much more rapidly than Δ*E*_Abs_ with increasing pressure. The PL line shape also changes with pressure significantly, unlike the exciton absorption peak, which is also reflected in the large changes in FWHM. The large disparity in the pressure responses of the absorbing exciton transition and emitting transitions contrasts with the case of CsPbBr_3_ QDs where both the absorbing and emitting transitions originate from the same exciton.

We attribute the observed PL in CsPbBr_3_ NPLs to trapped excitons at the sites created under pressure. The large Stokes shift and the broader bandwidth compared to the PL from the usual bandedge exciton of CsPbBr_3_ NPLs are characteristics of the trapped exciton PL observed in several MHP systems under ambient conditions, including certain 2D-layered structures,^27^ double perovskites,^28^ and MHPs doped with other metal ions.^29^ In the case of CsPbBr_3_ NPLs under ambient conditions, no trap-state PL with such a large Stokes shift has been previously observed, although the existence of a nonemitting shallow trap state 10 meV below the bandedge exciton was reported.^30^ The observation of the broad PL with a large Stokes shift indicates that pressure-induced creation of sufficiently deep trap states occurs at pressures as low as 0.1 GPa. We ruled out pressure-induced sintering of the NPLs to thicker NPLs that would exhibit exciton absorption and PL spectra at longer wavelengths due to the reduced quantum confinement.^20^ Although the pressure-induced sintering of CsPbBr_3_ NCs has been previously reported, it was observed only in a preassembled superlattice form.^11^ Furthermore, the reversible appearance of the exciton absorption peak at 440 nm attributed to 2 nm thick NPLs upon decompression further supports the absence of the pressure-induced sintering of NPLs.

**Figure 4:**
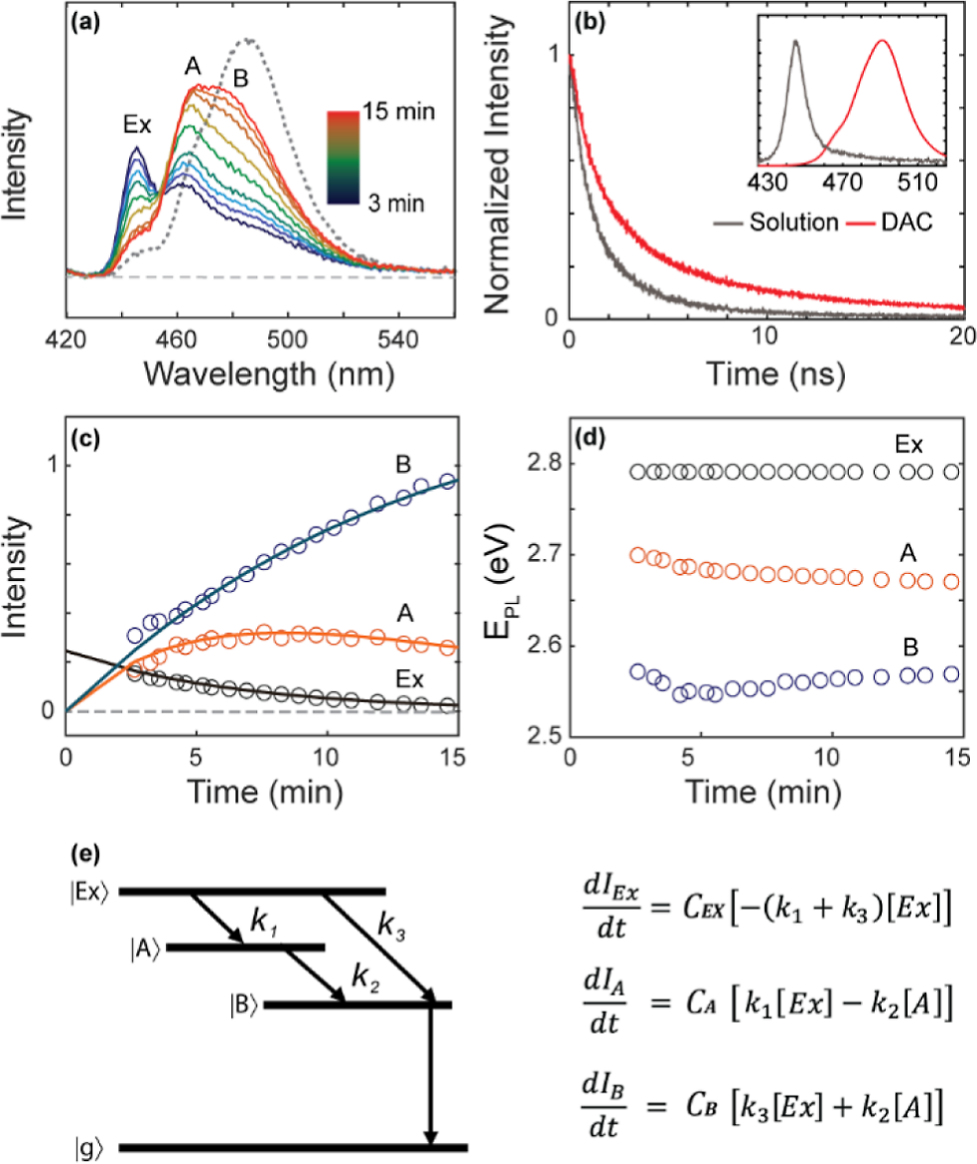
(a) Time-dependent PL from CsPbBr_3_ NPLs after setting the pressure to 0.1 GPa (ODE was used as the pressure-transmitting medium). The data could not be obtained during the first several minutes after the pressure. Ex: band-edge exciton PL; A and B: trapped exciton PL. (b) Comparison of the time-resolved PL intensities of the bandedge exciton PL centered at 445 nm and the red-shifted PL (peaks A and B combined). (c,d) Time-dependent peak intensities (c) and peak positions (d) of Ex, A, and B extracted from Figure 4a. The curves superimposed on panel (c) are from the kinetic modeling. (e) Kinetic model used to fit the time-dependent PL intensities shown in panel (c).

Because the remnant pressure remains greater than ambient conditions even upon full decompression of the DAC based on the observed ruby fluorescence, the reversibility of the pressure-induced trap creation in CsPbBr_3_ NPLs could not be determined unambiguously from the PL data in Figure 2d. An important clue to answering this question and additional insights into the intriguing low-pressure PL of CsPbBr_3_ NPLs were obtained from the following two experiments. First, we monitored the time-dependent PL spectra of CsPbBr_3_ NPLs at a pressure of 0.1 GPa, which is the first pressure point during the compression in the DAC. In Figure 4a, the spectral evolution during the first 15 min is shown as the solid curves. The dotted curve is the steady-state PL at 0.1 GPa obtained after ∼30 min, which did not change further even after waiting for a much longer time. Since following the initial pressurization of the DAC, inspection of the loaded sample, and placing the DAC in a microscope for the PL measurement took several minutes, the spectral change during this initial period could not be recorded. Figure 4a shows the rise of the broad red-shifted PL over time that can be decomposed into two peaks indicated as A and B at the expense of the intensity from the exciton PL (Ex) centered at 445 nm (2.79 eV). The time-dependent PL intensities and peak positions of all three peaks are shown in Figure 4c,d. Although all three peaks are already present at the first recorded time point (2.5 min) in Figure 4a, we confirmed that only Ex is present immediately after loading the sample in the DAC from a separate measurement. However, the initial Ex intensity value from this measurement is not included in Figure 4c because of its incompatibility with the rest of the PL measurements made on a microscope.

Despite the incompleteness of the time-dependent intensity data at the early time points, the resemblance of the time-dependent PL intensities of Ex (*I*_Ex_), A (*I*_A_), and B (*I*_B_) to the kinetics of the sequential transfer of the Ex population to A and B is apparent. Therefore, we attempted to fit the data to a simple kinetic model illustrated in Figure 4e to extract the rate information on the transformation of the bandedge exciton PL to trapped exciton PL. The following assumptions were made in this analysis to solve the rate equations shown in Figure 4e: (1) Ex can transform into A and B with rate constants *k*_1_ and *k*_3_, respectively, and A can transform into B with rate constant *k*_2_. (2) *I*_Ex_, *I*_A_, and *I*_B_ are proportional to the population of each state with different prefactors (*C*_Ex_, *C*_A_, and *C*_B_), reflecting the state-dependent emission quantum yield and transition cross section. Within the assumptions and limitations mentioned above, the data fit best to the model with the rate constants *k*_1_ = 1/13.3 (min^–1^), *k*_2_ = 1/10.8 (min^–1^), and *k*_3_ = 1/12.8 (min^–1^). The kinetic analysis indicates that the initial exciton transforms into two trapped states on a time scale of ∼10 min under a static pressure of 0.1 GPa. In Figure 4d, the A and B peaks show a small but continuing shift on the time scale of ∼10 min, whereas the Ex peak position remains constant. This suggests that the traps created under a constant pressure of 0.1 GPa continue to evolve until a steady state is reached after ∼30 min. The comparison of the PL lifetimes under the ambient pressure and at 0.1 GPa shown in Figure 4b reveals that the red-shifted PL has a longer lifetime (τ = 4.6 ns) than that of the exciton PL (τ = 3.5 ns), which is consistent with the expectation from the trapped exciton.^27^ Therefore, we interpret the spectral evolution of the PL at 0.1 GPa as resulting from the pressure-induced formation of the trap states A and B via local structural distortion, although their exact nature cannot be determined in this study. During the time window, the PL showed a large spectral evolution at 0.1 GPa, and the absorption spectrum of CsPbBr_3_ NPLs maintained the initial exciton absorption peak with little change. This disparity indicates that the structural changes responsible for the trapped exciton PL have a negligible effect on the absorbing exciton transition in NPLs, which is consistent with local structural distortions rather than a homogeneous structural change in the entire NPL.

In contrast to the large time dependence of the PL at 0.1 GPa discussed above, the PL at the higher pressure showed little time dependence. Above ∼0.2 GPa, the PL spectra exhibited only a small spectral shift (1 meV) without changing the spectral shape over the period of 10 min. Therefore, we conclude that the most significant structural change responsible for the trapped exciton PL occurs at the low-pressure regime. The subsequent increase in the pressure only gradually red-shifts the energy of the exciton absorption and trapped exciton PL until both the exciton absorption peak and PL disappear at >2 GPa. The trapped exciton PL of CsPbBr_3_ NPLs triggered at low pressure, unlike in CsPbBr_3_ QDs that do not show any trapped exciton PL, may be due to the smaller number of [PbBr_6_]^4–^ octahedral units (three layers) along the thickness direction that are more susceptible to the pressure-induced structural distortion. Especially when two of the three layers interface with the organic ligands, higher sensitivity to the pressure-induced structural distortion than that in the QDs with a larger number of unit cells may not be surprising. A systematic thickness-dependent study of the PL from CsPbBr_3_ NPLs under pressure will provide further insights into the pressure-induced trapped exciton PL.

**Figure 5:**
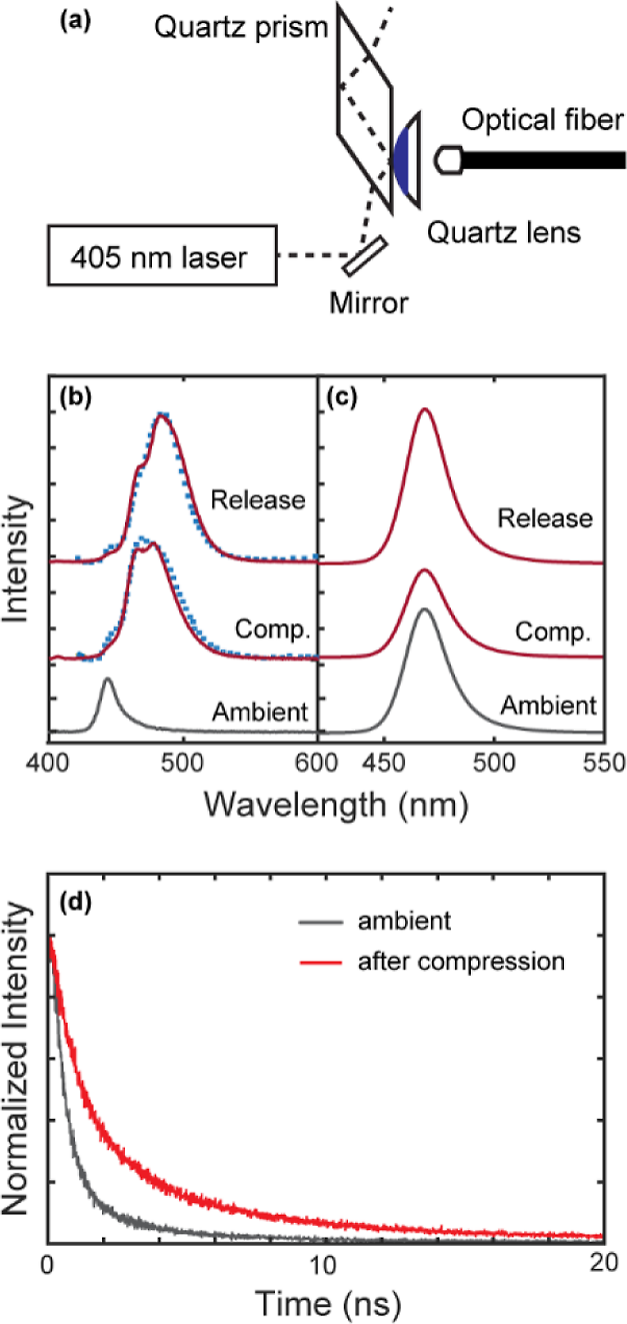
Measurement of the PL from the films of CsPbBr_3_ NCs compressed between a prism and a lens. (a) Schematic diagram of the experimental setup employing the attenuated total internal reflection excitation. The dashed line shows the beam path of the excitation light. The dark region of the lens indicates where the NC film is coated. (b,c) Comparison of the PL spectra of the films of (b) CsPbBr_3_ NPLs and (c) CsPbBr_3_ QDs deposited on the lens at ambient pressure, under compression, and full release of the pressure. The dotted curves in panel (b) superimposed on the PL under compression and release are the PL spectra measured in a DAC at 0.1 GPa shown in Figure 4a at ∼15 and ∼30 min, respectively. (d) Comparison of the time-resolved PL intensity from CsPbBr_3_ NPLs before and after the compression.

A second experiment was performed to obtain additional information regarding the irreversibility of trap creation in CsPbBr_3_ NCs at low pressure. To directly probe the spectral change upon the return of the system fully to ambient pressure without any remnant pressure, we constructed the setup shown in Figure 5a. Here, the pressure on CsPbBr_3_ NCs is created by sandwiching the film of CsPbBr_3_ NCs between the two quartz substrates, one flat (prism) and the other curved (lens). Separating the prism and lens fully releases the pressure to the ambient conditions unlike in the DAC. A film of CsPbBr_3_ NCs was formed on the surface of the convex side of the quartz lens. In this setup, only the part of the NC film at the contact point of the prism and lens experiences the applied pressure. The photoexcitation of only the pressurized part of the NC film was achieved via attenuated total internal reflection at the contact point of the prism and lens by adjusting the incident angle of the laser light to be larger than the critical angle. The pressure was created by gently pushing the lens against the prism using a spring-loaded linear translation stage, on which the lens was mounted. An optical fiber was used to collect PL for measurement of the spectrum and PL lifetime. Although the pressure exerted on the sample at the contact point of the prism and lens cannot be readily estimated, it should be significantly lower than the compressive strength of the quartz lens, ∼1 GPa. We estimate that we are firmly in a low-pressure regime comparable to the lowest-pressure data points collected in the DAC based on similarities in the observed spectra (see below).

Figure 5b,c compares the PL spectra of the films of CsPbBr_3_ NPLs and QDs compressed at the contact point of the prism and lens and after the release of the pressure. The spectra of the NC films under ambient pressure are also shown for comparison. In contrast to the film of CsPbBr_3_ QDs, whose PL peak positions are not changing, the film of CsPbBr_3_ NPLs exhibits a significantly red-shifted PL upon compression. The PL spectrum of CsPbBr_3_ NPL under compression is very similar to the spectra obtained in a DAC under 0.1 GPa at a time of 15 min, as shown in Figure 4a. The lifetime of the red-shifted trapped exciton PL under compression shown in Figure 5d increased to 4.3 from 2.8 ns of exciton PL at the ambient pressure, similarly to the comparison made in Figure 4b in DAC. Interestingly, the PL spectrum upon returning to the ambient pressure did not recover the initial exciton PL. It is closer to the steady-state spectrum in DAC at 0.1 GPa achieved after ∼30 min. These observations indicate that the structural distortion responsible for the trapped exciton PL in the CsPbBr_3_ NPL is irreversible and can be triggered at relatively low pressures such as those created at the contact points of macroscopic solid surfaces. This has an important implication when using these MHP NPLs for light-emitting applications on a platform that can potentially impose comparable pressures on the NPLs to produce trap states. Modifying the surface-bound ligand or substitutionally doping the cation sites of the lattice in ways that have been shown to increase the structural stability of MHP NCs will be possible strategies to alter the behavior of the PL at low pressures in future studies.^31,32^

## Conclusions

We investigated the effects of the pressure on the exciton transitions in strongly quantum-confined CsPbBr_3_ QDs and NPLs, each representing two common morphologies of semiconductor NCs with a confined exciton in 0D and 2D structures. Taking advantage of the well-isolated exciton peak from the continuum interband absorption, we revealed the pressure-induced switching off and on of the exciton transition previously not resolvable in nonconfined NCs and the high susceptibility of CsPbBr_3_ NPLs to pressure-induced trap creation. In CsPbBr_3_ QDs, both the absorbing and emitting exciton transitions originate from the same band edge exciton without exhibiting any signature of trap states. The exciton absorption and PL peaks showed a continuous red shift with increasing pressure until the exciton transition peak vanished above ∼1.5 GPa, accompanied by the quenching of the PL in a reversible manner. CsPbBr_3_ NPLs also showed reversible pressure-induced switching off and on of the exciton transition similarly to CsPbBr_3_ QDs. However, the PL of CsPbBr_3_ NPLs originated from trap states created irreversibly even at the lowest pressure in this study (0.1 GPa), while the absorbing exciton transition was from the band edge exciton. Time-dependent PL measurements indicated that the creation of the trap states in CsPbBr_3_ NPLs under static pressures of 0.1 GPa occurred on the time scale of ∼10 min, which may reflect the overall softer structure of the NPLs than that of the QDs.

## Supporting Information Available

Full pressure-dependent PL and absorption spectra of CsPbBr_3_ QDs and NPLs, comparison of the PL peak shift and PL line width in different pressure-transmitting media, and comparison of the absorption spectra of CsPbBr_3_ NCs in different pressure-transmitting media (PDF)

## References

[ref1] Tolbert S. H.; Herhold A. B.; Johnson C. S.; Alivisatos A. P. Comparison of quantum confinement effects on the electronic absorption spectra of direct and indirect gap semiconductor nanocrystals. Phys. Rev. Lett. 1994, 73 (24), 3266–3269. 10.1103/PhysRevLett.73.3266. 10057333

[ref2] Choi C. L.; Koski K. J.; Sivasankar S.; Alivisatos A. P. Strain-dependent photoluminescence behavior of CdSe/CdS nanocrystals with spherical, linear, and branched topologies. Nano Lett. 2009, 9 (10), 3544–3549. 10.1021/nl9017572. 19678687 PMC2768666

[ref3] Meulenberg R. W.; Strouse G. F. Pressure-induced electronic coupling in CdSe semiconductor quantum dots. Phys. Rev. B 2002, 66 (3), 035317 10.1103/PhysRevB.66.035317.

[ref4] Tolbert S. H.; Alivisatos A. P. High-pressure structural transformations in semiconductor nanocrystals. Annu. Rev. Phys. Chem. 1995, 46, 595–626. 10.1146/annurev.pc.46.100195.003115. 24341343

[ref5] Meng L.; Lane J. M. D.; Baca L.; Tafoya J.; Ao T.; Stoltzfus B.; Knudson M.; Morgan D.; Austin K.; Park C.; et al. Shape Dependence of Pressure-Induced Phase Transition in CdS Semiconductor Nanocrystals. J. Am. Chem. Soc. 2020, 142 (14), 6505–6510. 10.1021/jacs.0c01906. 32202423 PMC7786387

[ref6] Wang Z.; Wen X.-D.; Hoffmann R.; Son J. S.; Li R.; Fang C. C.; Smilgies D.-M.; Hyeon T. Reconstructing a solid-solid phase transformation pathway in CdSe nanosheets with associated soft ligands. Proc. Natl. Acad. Sci. 2010, 107 (40), 17119–17124. 10.1073/pnas.1011224107. 20855580 PMC2951424

[ref7] Meng L.; Fan H.; Lane J. M.; Baca L.; Tafoya J.; Ao T.; Stoltzfus B.; Knudson M.; Morgan D.; Austin K.; et al. X-Ray Diffraction and Electron Microscopy Studies of the Size Effects on Pressure-Induced Phase Transitions in CdS Nanocrystals. MRS Adv. 2020, 5 (48–49), 2447–2455. 10.1557/adv.2020.191.

[ref8] Pan Y.; Qu S.; Dong S.; Cui Q.; Zhang W.; Liu X.; Liu J.; Liu B.; Gao C.; Zou G. An investigation on the pressure-induced phase transition of nanocrystalline ZnS. J. Phys.: Condens. Matter 2002, 14 (44), 10487–10490. 10.1088/0953-8984/14/44/320.

[ref9] Chen Z.; Beimborn J. C.; Kirkwood N.; Russo S. P.; Weber J. M.; Mulvaney P. Size-Dependent Response of CdSe Quantum Dots to Hydrostatic Pressure. J. Phys. Chem. C 2023, 127 (18), 8657–8669. 10.1021/acs.jpcc.3c00998.

[ref10] Xiao G.; Cao Y.; Qi G.; Wang L.; Liu C.; Ma Z.; Yang X.; Sui Y.; Zheng W.; Zou B. Pressure Effects on Structure and Optical Properties in Cesium Lead Bromide Perovskite Nanocrystals. J. Am. Chem. Soc. 2017, 139 (29), 10087–10094. 10.1021/jacs.7b05260. 28682634

[ref11] Nagaoka Y.; Hills-Kimball K.; Tan R.; Li R.; Wang Z.; Chen O. Nanocube Superlattices of Cesium Lead Bromide Perovskites and Pressure-Induced Phase Transformations at Atomic and Mesoscale Levels. Adv. Mater. 2017, 29 (18), 1606666 10.1002/adma.201606666. 28295682

[ref12] Beimborn J. C.; Walther L. R.; Wilson K. D.; Weber J. M. Size-Dependent Pressure-Response of the Photoluminescence of CsPbBr_3_ Nanocrystals. J. Phys. Chem. Lett. 2020, 11 (5), 1975–1980. 10.1021/acs.jpclett.0c00174. 32066242

[ref13] Jing X.; Sun R.; Tian H.; Liu R.; Liu B.; Zhou D.; Li Q.; Liu B. Evolution of self-trapped exciton emission tuned by high pressure in 2D all-inorganic cesium lead halide nanosheets. J. Mater. Chem. C 2022, 10 (22), 8711–8718. 10.1039/D2TC01465C.

[ref14] Ma Z.; Liu Z.; Lu S.; Wang L.; Feng X.; Yang D.; Wang K.; Xiao G.; Zhang L.; Redfern S. A. T.; et al. Pressure-induced emission of cesium lead halide perovskite nanocrystals. Nat. Commun. 2018, 9 (1), 4506 10.1038/s41467-018-06840-8. 30374042 PMC6206024

[ref15] Yin T.; Liu B.; Yan J.; Fang Y.; Chen M.; Chong W. K.; Jiang S.; Kuo J. L.; Fang J.; Liang P.; et al. Pressure-Engineered Structural and Optical Properties of Two-Dimensional (C_4_H_9_NH_3_)_2_PbI_4_ Perovskite Exfoliated nm-Thin Flakes. J. Am. Chem. Soc. 2019, 141 (3), 1235–1241. 10.1021/jacs.8b07765. 30561996

[ref16] Muscarella L. A.; Dučinskas A.; Dankl M.; Andrzejewski M.; Casati N. P. M.; Rothlisberger U.; Maier J.; Graetzel M.; Ehrler B.; Milić J. V. Reversible Pressure-Dependent Mechanochromism of Dion-Jacobson and Ruddlesden-Popper Layered Hybrid Perovskites. Adv. Mater. 2022, 34 (17), e2108720 10.1002/adma.202108720. 35181967

[ref17] Mączka M.; Sobczak S.; Ratajczyk P.; Leite F. F.; Paraguassu W.; Dybała F.; Herman A. P.; Kudrawiec R.; Katrusiak A. Pressure-Driven Phase Transition in Two-Dimensional Perovskite MHy_2_PbBr_4_. Chem. Mater. 2022, 34 (17), 7867–7877. 10.1021/acs.chemmater.2c01533.

[ref18] Dong Y.; Qiao T.; Kim D.; Parobek D.; Rossi D.; Son D. H. Precise Control of Quantum Confinement in Cesium Lead Halide Perovskite Quantum Dots via Thermodynamic Equilibrium. Nano Lett. 2018, 18 (6), 3716–3722. 10.1021/acs.nanolett.8b00861. 29727576

[ref19] Cheng O. H.; Qiao T.; Sheldon M.; Son D. H. Size- and temperature-dependent photoluminescence spectra of strongly confined CsPbBr_3_ quantum dots. Nanoscale 2020, 12 (24), 13113–13118. 10.1039/D0NR02711A. 32584332

[ref20] Dong Y.; Qiao T.; Kim D.; Rossi D.; Ahn S. J.; Son D. H. Controlling Anisotropy of Quantum-Confined CsPbBr_3_ Nanocrystals by Combined Use of Equilibrium and Kinetic Anisotropy. Chem. Mater. 2019, 31 (15), 5655–5662. 10.1021/acs.chemmater.9b01515.

[ref21] Piermarini G. J.; Block S.; Barnett J. D.; Forman R. A. Calibration of the pressure dependence of the R1 ruby fluorescence line to 195 kbar. J. Appl. Phys. 1975, 46 (6), 2774–2780. 10.1063/1.321957.

[ref22] Mao H. K.; Xu J.; Bell P. M. Calibration of the ruby pressure gauge to 800 kbar under quasi-hydrostatic conditions. J. Geophys. Res.: Solid Earth 1986, 91 (B5), 4673–4676. 10.1029/JB091iB05p04673.

[ref23] Amat A.; Mosconi E.; Ronca E.; Quarti C.; Umari P.; Nazeeruddin M. K.; Gratzel M.; De Angelis F. Cation-induced band-gap tuning in organohalide perovskites: interplay of spin-orbit coupling and octahedra tilting. Nano Lett. 2014, 14 (6), 3608–3616. 10.1021/nl5012992. 24797342

[ref24] Klimov V. I. Nanocrystal quantum dots, 2nd ed.; CRC Press Boca Raton: Boca Raton, 2010.

[ref25] Wang Y.; Lü X.; Yang W.; Wen T.; Yang L.; Ren X.; Wang L.; Lin Z.; Zhao Y. Pressure-Induced Phase Transformation, Reversible Amorphization, and Anomalous Visible Light Response in Organolead Bromide Perovskite. J. Am. Chem. Soc. 2015, 137 (34), 11144–11149. 10.1021/jacs.5b06346. 26284441

[ref26] Tolbert S. H.; Alivisatos A. P. Size Dependence of a First Order Solid-Solid Phase Transition: The Wurtzite to Rock Salt Transformation in CdSe Nanocrystals. Science 1994, 265 (5170), 373–376. 10.1126/science.265.5170.373. 17838040

[ref27] Liang M.; Lin W.; Zhao Q.; Zou X.; Lan Z.; Meng J.; Shi Q.; Castelli I. E.; Canton S. E.; Pullerits T.; et al. Free Carriers versus Self-Trapped Excitons at Different Facets of Ruddlesden-Popper Two-Dimensional Lead Halide Perovskite Single Crystals. J. Phys. Chem. Lett. 2021, 12 (20), 4965–4971. 10.1021/acs.jpclett.1c01148. 34014103 PMC8279734

[ref28] Wu B.; Ning W.; Xu Q.; Manjappa M.; Feng M.; Ye S.; Fu J.; Lie S.; Yin T.; Wang F.; et al. Strong self-trapping by deformation potential limits photovoltaic performance in bismuth double perovskite. Sci. Adv. 2021, 7 (8), eabd3160 10.1126/sciadv.abd3160. 33597239 PMC7888938

[ref29] Ma Z.; Li Q.; Luo J.; Li S.; Sui L.; Zhao D.; Yuan K.; Xiao G.; Tang J.; Quan Z.; et al. Pressure-Driven Reverse Intersystem Crossing: New Path toward Bright Deep-Blue Emission of Lead-Free Halide Double Perovskites. J. Am. Chem. Soc. 2021, 143 (37), 15176–15184. 10.1021/jacs.1c06207. 34506135

[ref30] Socie E.; Vale B. R. C.; Burgos-Caminal A.; Moser J.-E. Direct Observation of Shallow Trap States in Thermal Equilibrium with Band-Edge Excitons in Strongly Confined CsPbBr_3_ Perovskite Nanoplatelets. Adv. Opt. Mater. 2020, 9 (1), 2001308 10.1002/adom.202001308.

[ref31] Krieg F.; Ong Q. K.; Burian M.; Raino G.; Naumenko D.; Amenitsch H.; Süess A.; Grotevent M. J.; Krumeich F.; Bodnarchuk M. I.; et al. Stable Ultraconcentrated and Ultradilute Colloids of CsPbX(3) (X = Cl, Br) Nanocrystals Using Natural Lecithin as a Capping Ligand. J. Am. Chem. Soc. 2019, 141 (50), 19839–19849. 10.1021/jacs.9b09969. 31763836 PMC6923794

[ref32] Zhang Y.; Hou G.; Wu Y.; Chen M.; Dai Y.; Liu S.; Zhao Q.; Lin H.; Fang J.; Jing C.; et al. Surface Reconstruction of CsPbBr(3) Nanocrystals by the Ligand Engineering Approach for Achieving High Quantum Yield and Improved Stability. Langmuir 2023, 39 (17), 6222–6230. 10.1021/acs.langmuir.3c00393. 37079335

