# Supplementary material for: Effects of Pressure on Exciton Absorption and Emission in Strongly Quantum-Confined CsPbBr3 Quantum Dots and Nanoplatelets
Source: J Phys Chem C Nanomater Interfaces. 2024 Jan 31;128(5):2062–9. doi: 10.1021/acs.jpcc.3c08029 (PMC10860125; doi:10.1021/acs.jpcc.3c08029)
Supplement: Supplementary file 1 — jp3c08029_si_001.pdf [file jp3c08029_si_001.pdf]

Supplementary Information for

Effects of Pressure on Exciton Absorption and Emission in Strongly Quantum-confined $CsPbBr_3$ Quantum Dots and Nanoplatelets

Chih-Wei Wang[1], Ebube E. Oyeka[1], Alison B. Altman[1,*], Dong Hee Son[1,2,3*]

[1]Department of Chemistry, Texas A&M University, College Station, Texas 77843, United States

[2]Department of Physics and Astronomy, Texas A&M University, College Station, Texas 77843, United States

[3]Center for Nanomedicine, Institute for Basic Science and Graduate Program of Nano Biomedical Engineering, Advanced Science Institute, Yonsei University, Seoul 03722, Republic of Korea

Chemicals

Cesium carbonate (99.994%, Alfa Aesar), lead bromide (98%, Alfa Aesar), copper (II) bromide (97%, Sigma Aldrich), cobalt (II) bromide (98%, Alfa Aesar), zinc bromide (99%, BeanTown Chemical), didodecyldimethylammonium bromide (98%, TCI), 1-octadecene (90% technical grade, Sigma Aldrich), oleic acid (90% technical grade, Sigma Aldrich) and silicone oil (For oil baths, Sigma Aldrich) were used without further purification. Oleylamine (≥98%, Sigma Aldrich) was purified following the protocol described by Baranov, et al. [1] before use.

Additional Figures

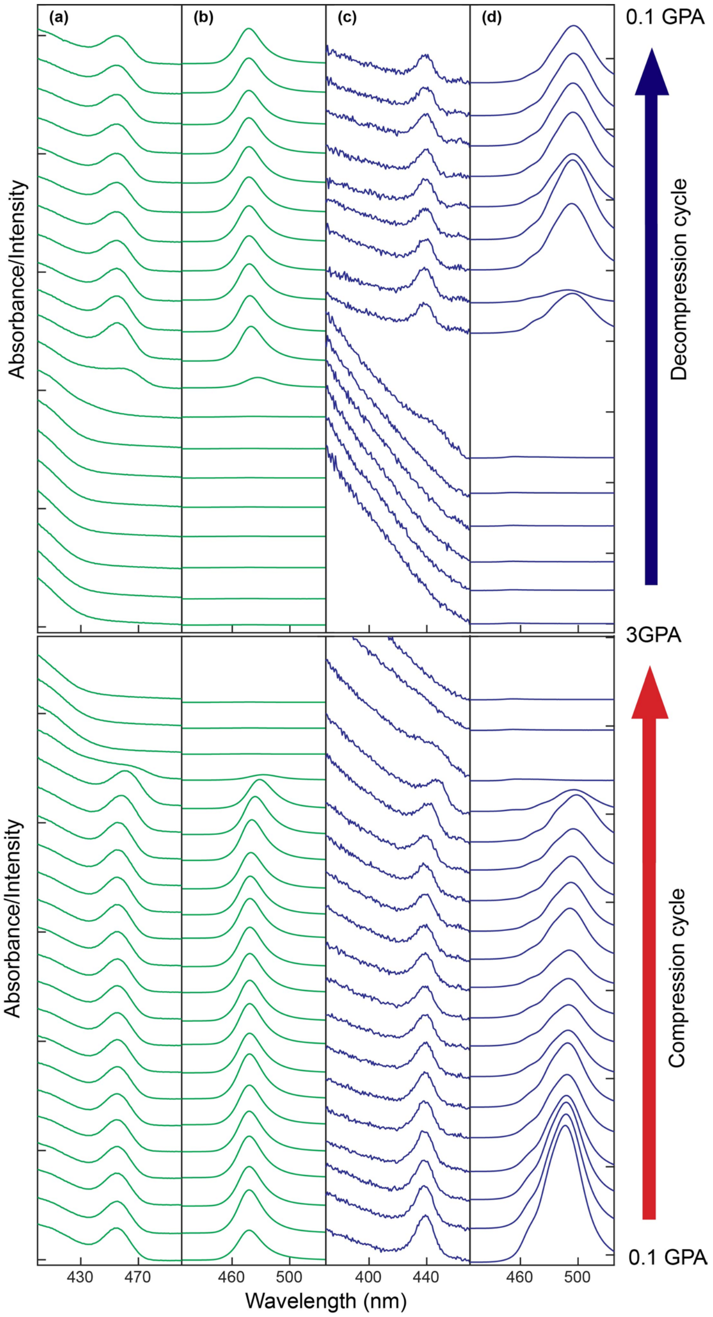

Figure S1. A more extensive set of pressure-dependent absorption and PL spectra of $CsPbBr_3$ NCs during the compression and decompression cycle. (a) Absorption spectra of $CsPbBr_3$ QDs (b) PL spectra of $CsPbBr_3$ QDs. (c) Absorption spectra of $CsPbBr_3$ NPLs. (d) PL spectra of $CsPbBr_3$ QNPLs. ODE was used as the pressure transmitting medium.

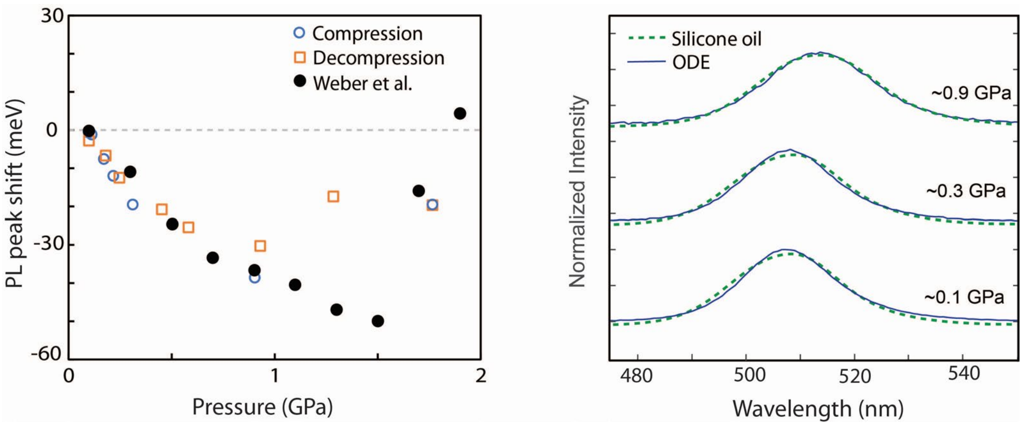

Figure S2. (a) Comparison of the pressure-dependent PL peak shift of ~8 nm $CsPbBr_3$ QDs in our study performed using octadecene (ODE) and the data in the literature performed in paraffin oil using 7.9 nm $CsPbBr_3$ QDs by Weber and coworkers[S2]. Data from our study and ref. S2 show very similar PL peak shift at varying pressures except one point on the decompression cycle near 1.3 GPa. (b) Comparison of PL spectral lineshapes of exciton PL measured from ~ 8nm $CsPbBr_3$ QDs in silicone oil and ODE at several pressures. Spectral lineshape and the linewidth of the PL in both pressure transmitting media. The similarity of the pressure-dependent PL peak shift and PL linewidth between ODE and silicone oil (or paraffin oil) considered to produce the hydrostatic pressure indicates that ODE is a viable pressure-transmitting medium that also provides the required solubility of the samples in this study.

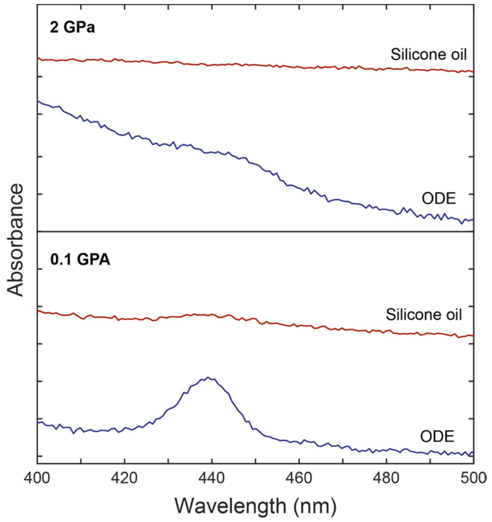

Figure S3. Comparison of the absorption spectra of $CsPbBr_3$ NPLs ODE (solid) and silicone oil (dashed). The cause of the aggregation that becomes more severe with increasing pressure, the reliable measurement of the pressure response of the exciton absorption was difficult in silicone oil.

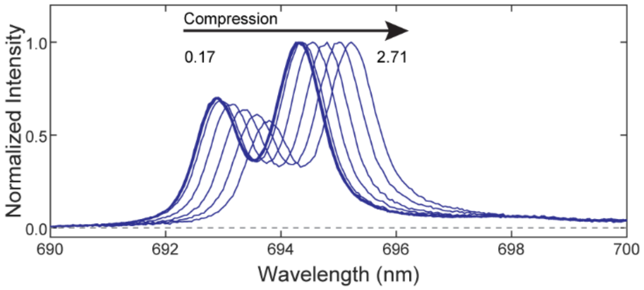

Figure S4. Evolution of ruby emission during typical compression cycle. Number indicates the pressure in GPa. ODE was used as the pressure transmitting medium.

References

S1. Baranov, D.; Lynch, M. J.; Curtis, A. C.; Carollo, A. R.; Douglass, C. R.; Mateo-Tejada, A. M.; Jonas, D. M., Purification of Oleylamine for Materials Synthesis and Spectroscopic Diagnostics for trans Isomers. *Chem. Mater.* **2019,** *31* (4), 1223-1230.

S2. Beimborn, J. C., 2nd; Walther, L. R.; Wilson, K. D.; Weber, J. M., Size-Dependent Pressure-Response of the Photoluminescence of $CsPbBr_3$ Nanocrystals. *J Phys Chem Lett* **2020,** *11* (5), 1975-1980.
